# Skin barrier dysfunction and correlation with the onset and progression of psoriasis

**DOI:** 10.3389/fimmu.2026.1808904

**Published:** 2026-06-30

**Authors:** Ying Wang, Yanyang Liang, Jianxiao Xing, Junqin Li, Kaiming Zhang

**Affiliations:** ShanXi Key Laboratory of Stem Cells for Immunological Dermatosis, State Key Breeding Laboratory of Stem Cells for Immunological Dermatosis, Institute of Dermatology, Taiyuan Central Hospital of Shanxi Medical University, Taiyuan, China

**Keywords:** correlation, onset, progression, psoriasis, skin barrier

## Abstract

The skin barrier serves as the body’s primary line of defense against exogenous insults and is critical for maintaining systemic homeostasis. Emerging evidence indicates that skin barrier dysfunction is not merely a secondary manifestation of psoriasis, but rather an early and pivotal pathogenic driver of the disease. It exerts bidirectional crosstalk with immune dysregulation, genetic predisposition and environmental triggers, collectively contributing to the initiation and progression of psoriasis. This review first elaborates on the normal structural composition and physiological functions of the skin barrier, followed by a discussion of the major etiological factors of psoriasis and the underlying mechanisms by which barrier impairment promotes psoriasis pathogenesis. These mechanisms include aberrant keratinocyte hyperproliferation, abnormal expression of structural proteins, tight junction disruption, aquaporin 3 dysfunction and dysregulated cutaneous lipid metabolism. Furthermore, we highlight the clinical significance of skin barrier restoration in psoriasis management and propose directions for future research. Elucidating the intricate crosstalk between skin barrier dysfunction and psoriasis pathogenesis will facilitate the identification of novel diagnostic biomarkers and the development of targeted therapeutic strategies for this disease.

## 1 Introduction

Psoriasis is a common chronic inflammatory autoimmune skin disease with a global prevalence of 0.1% to 3%, imposing a heavy physical and psychological burden on patients. Clinically, it is characterized by well-demarcated erythematous plaques covered with silvery-white scales and is mainly classified into four subtypes, with plaque psoriasis being the most prevalent. Despite advances in therapeutic strategies, including immunosuppressants, biological agents, and phototherapy, the disease remains associated with a high recurrence rate, and some patients exhibit treatment resistance-particularly those with lesions in areas with inherently poor skin barrier function. The long-term disease course and visible cutaneous lesions often induce psychological distress, severely impairing patients’ quality of life, which highlights the urgency of clarifying its pathogenesis and exploring novel therapeutic targets (1, 2).

As the largest organ of the human body, the skin’s barrier function serves as the foundation for maintaining homeostasis. It forms a complex integrated system comprising the stratum corneum (analogized to the “brick-and-mortar” structure, where keratinocytes act as “bricks” and intercellular lipids as “mortar”), tight junctions, skin microflora, and immune/chemical barriers. This system defends against external insults, prevents water loss, and regulates immune responses. Accumulating evidence confirms that skin barrier dysfunction is closely associated with the onset and progression of psoriasis, functioning as an important initiating factor rather than a secondary manifestation (3, 4).

Despite the growing body of research on their correlation, significant gaps persist: the core molecular mechanisms underlying their interaction remain unclear; findings on key barrier-related molecules (e.g., aquaporin 3, tight junction proteins) in psoriatic lesions are inconsistent; the clinical application of skin barrier repair is not standardized; and the correlation between barrier evaluation indicators and disease severity, treatment response, or prognosis has not been fully elucidated (5). To address these research gaps, this review systematically summarizes the research progress regarding the correlation between skin barrier dysfunction and psoriasis, elaborates on their interaction mechanisms, analyzes the clinical value of skin barrier repair, and identifies existing research deficiencies and future research directions (6). It aims to clarify the core role of skin barrier dysfunction in psoriasis, provide a theoretical basis for clinical diagnosis and treatment, and offer new insights into targeted therapeutic strategies (7, 8).

## 2 Review methodology

This review was performed to summarize the current evidence regarding skin barrier dysfunction in psoriasis, with a focus on keratin structure, filaggrin-related proteins, tight junctions, aquaporin 3, and skin lipid metabolism. Relevant studies were identified through systematic searches in PubMed, Web of Science, Embase, and CNKI databases from their inception up to February 2026. The search strategy combined key terms including “psoriasis” “skin barrier” “epidermal permeability barrier” “keratin” “filaggrin” “tight junction” “aquaporin 3” “Lipid metabolism” “ratum corneum hydration” and “transepidermal water loss”.

Only peer-reviewed full-text articles published in English or Chinese were considered. Original research articles, clinical trials, and high-quality reviews investigating structural, functional, or molecular abnormalities of the skin barrier in psoriasis were included. Studies focusing exclusively on *in vitro* models, animal experiments without clinical correlation, case reports, meeting abstracts, and duplicate publications were excluded.

Two authors independently screened titles, abstracts, and full texts to assess eligibility. Discrepancies were resolved through discussion until consensus was reached. Key data extracted included study design, patient population, main barrier-related indicators (such as TEWL, SCH, lipid composition, protein expression levels), molecular mechanisms, and therapeutic implications for barrier repair. All extracted information was synthesized narratively to outline the roles of multiple structural and functional components in psoriasis-related barrier impairment, as well as their potential as therapeutic targets.

## 3 Physiological structure and function of the skin barrier

### 3.1 Structure of the skin barrier

**Figure 1:**
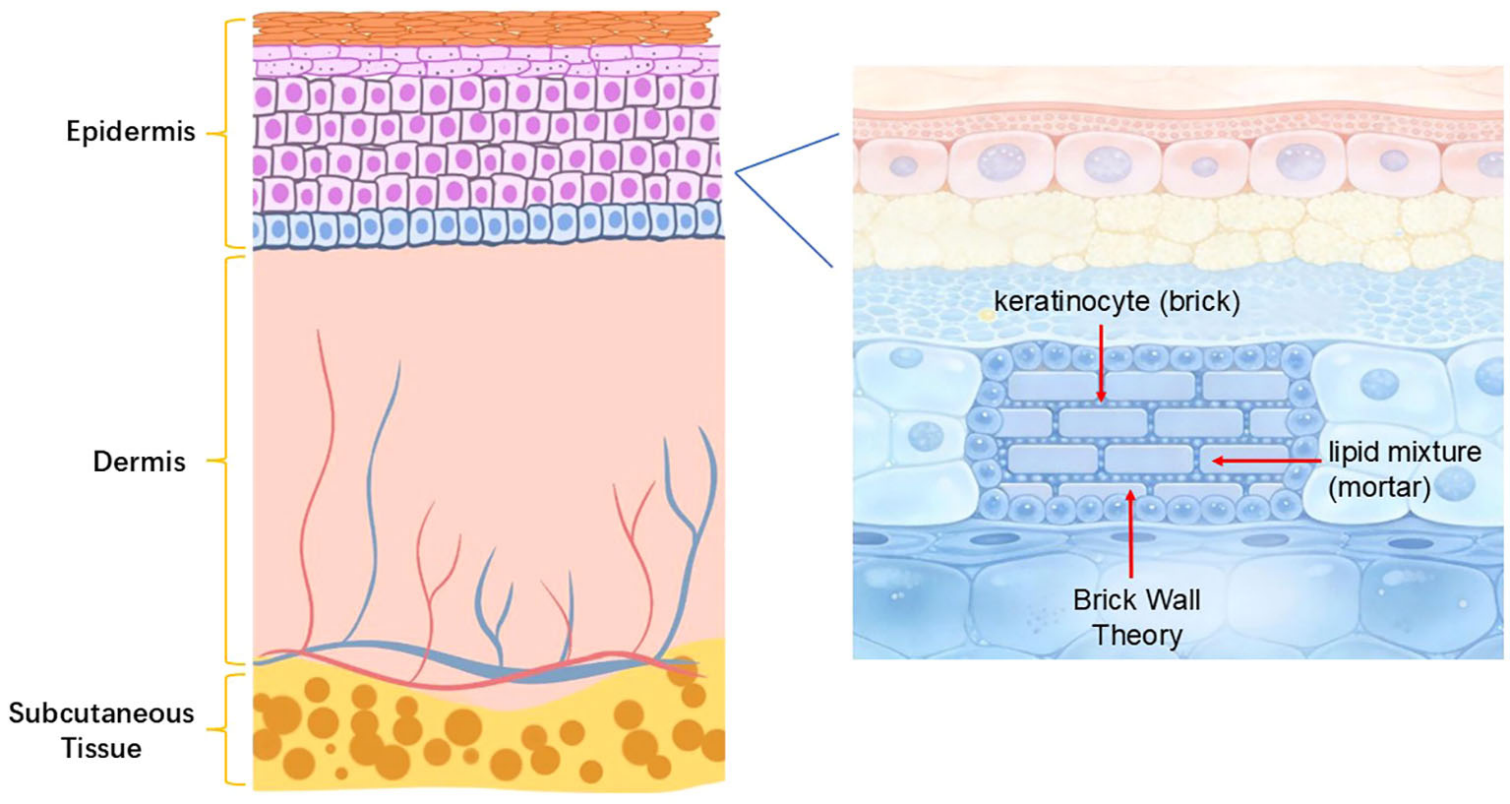
The brick-and-mortar structure of the skin barrier. The brick-and-mortar structural model of the skin barrier, with keratinocytes serving as structural “bricks” and intercellular lipid matrix as adhesive “mortar” to maintain barrier integrity.

The epidermal barrier constitutes the core component of the skin barrier. Structurally, it comprises five layers from the inside out: the stratum basale, stratum spinosum, stratum granulosum, stratum lucidum, and stratum corneum. Among these, the stratum granulosum and stratum corneum are critical for maintaining normal barrier function, with the stratum corneum serving as the fundamental structural basis of the epidermal barrier. The stratum corneum is a layered structure composed of fully differentiated keratinocytes (KCs) and intercellular lipid mixtures, acting as the body’s primary permeability barrier. This structural feature is consistent with the “brick-and-mortar” model proposed by Professor Peter in 1983 (9). In this model, keratinocytes are regarded as “bricks” and intercellular lipids as “mortar”; their tight integration prevents external invasion and limits excessive transepidermal water loss (10). The brick-and-mortar structure is illustrated in **Figure 1**.

Keratinocytes arise from the stratum basale, migrate upward, undergo differentiation, and ultimately form anucleated corneocytes, which represent the key components of the epidermal barrier. Intracellular keratin and filaggrin assemble into fibrous bundles that are essential for barrier stability and resistance to mechanical damage (11). Mature keratinocytes form an extracellular cornified envelope and lipid membrane, which function as a protective layer. Corneocytes contain keratin and natural moisturizing factors (NMFs), and synthesize lipids as well as related enzymes (12). The intracellular compartment is encircled by an insoluble cornified envelope composed of loricrin, involucrin, and other proteins, which is indispensable for the barrier function of the stratum corneum (13, 14). Dysregulated expression or genetic mutations of these associated proteins can directly lead to epidermal barrier dysfunction. For example, loricrin-knockout mice display xerosis cutis and hyperkeratosis, typical manifestations of severe skin barrier impairment (13, 14). Aberrant loricrin expression can also trigger keratinization disorders and ichthyosis. In addition, filaggrin is proteolytically degraded to generate NMFs; reduced NMF levels compromise the structural integrity of the stratum corneum, a pathological change closely associated with the pathogenesis of atopic dermatitis.

As the “mortar” in the brick-and-mortar model, intercellular lipids represent key structural components of the epidermal barrier (14). They are mainly composed of ceramides (approximately 50%), cholesterol (approximately 25%), and free fatty acids (approximately 15%), with the remainder consisting of other lipids essential for sustaining the barrier function of the stratum corneum. Precursor substances of intercellular lipids are synthesized by spinous keratinocytes and stored in lamellar bodies, which are distributed in the cytoplasm of cells in the stratum spinosum and stratum granulosum (15). During keratinocyte differentiation and maturation, lamellar bodies release lipid precursors and related enzymes; subsequent enzymatic hydrolysis of these precursors produces intercellular structural lipids (16, 17). Ceramides play a central role in maintaining epidermal barrier stability, whereas free fatty acids and cholesterol also exert pivotal functions (18, 19). Dysfunction of lamellar bodies can cause abnormal skin desquamation (16, 17), whereas their massive secretion promotes barrier repair following acute skin injury (20). Furthermore, both dysregulation of intercellular lipid composition and lamellar body dysfunction can impair the epidermal barrier, thereby increasing susceptibility to inflammatory skin diseases such as atopic dermatitis (21).

Beyond the core brick-and-mortar architecture, corneodesmosomes and skin appendages further strengthen epidermal barrier function. Corneodesmosomes, derived from intercellular desmosomes during keratinocyte differentiation, mediate mechanical adhesion between corneocytes and regulate physiological skin desquamation (22). Abnormalities in their abundance or morphology are associated with desquamative disorders; for instance, psoriasis is linked to a shortened keratinocyte differentiation cycle and dysregulation of corneodesmosome-associated enzymes (23). Skin appendages act synergistically to reinforce the epidermal barrier: sebaceous glands secrete sebum that forms the core of the cutaneous chemical barrier, hair follicles participate in keratin repair and innate immune defense, and sweat glands regulate body temperature while stabilizing the sebaceous film.

### 3.2 Evaluation indicators for abnormal skin barrier function

Key indicators for evaluating abnormal skin barrier function encompass transepidermal water loss (TEWL), stratum corneum hydration (SCH), sebum content, skin surface pH, and epidermal thickness (24). As the most widely employed non-invasive quantitative indicator, TEWL serves as a reliable marker of epidermal permeability barrier integrity; barrier impairment elicits a marked elevation in TEWL, a characteristic hallmark of atopic dermatitis, psoriasis, and ichthyosis (24, 25). Patients with atopic dermatitis demonstrate an exaggerated TEWL increase in non-lesional skin following barrier challenge, indicating inherent fragility of the skin barrier (26). Complementary evaluative approaches include mass spectrometry-based lipid profiling, transmission electron microscopy, and immunofluorescence staining for barrier-associated proteins.

Reduced SCH (below 10%) denotes impaired water-holding capacity, which presents clinically as dryness, scaling, and pruritus (27). This reduction is prominent in atopic dermatitis and aging skin, associated with decreased lipid levels and filaggrin expression (26), and can be exacerbated by abnormal skin surface pH, over-cleansing, or the application of irritant topical products. SCH is indirectly assessed through skin conductivity measurements.

Sebum content is a critical marker reflecting the integrity of the skin surface lipid film. Excessive sebum secretion contributes to the pathogenesis of acne and is further exacerbated by treatment-related barrier injury (28), whereas insufficient secretion results in defective lipid films, a key feature of barrier impairment in atopic dermatitis and senile xerosis.

Notably, in psoriasis, lesional skin is characterized by elevated TEWL, increased erythema index, and reduced SCH, all of which are indicative of severe barrier disruption. These abnormalities can be partially reversed with clinical improvement (e.g., post-phototherapy), thereby facilitating the monitoring of disease activity and treatment efficacy.

## 4 The correlation mechanism between psoriasis and skin barrier

Abnormal skin barrier function is not a secondary manifestation following the onset of psoriasis, but a core link deeply involved in the occurrence and progression of the disease. This abnormality is closely associated with abnormal keratinocyte proliferation, immune dysfunction, aberrant expression of epidermal structural proteins, lipid metabolism disorders, and impaired aquaporin function. Collectively, these factors are intertwined and act synergistically to drive the development and chronicity of psoriasis.

### 4.1 Genetic and structural basis related to the barrier

Skin barrier defects in patients with psoriasis exhibit a significant genetic predisposition, involving multiple dimensions including epidermal structure, cell junctions, lipid metabolism, and hydration capacity, which provide congenital conditions for disease initiation. Genome-wide association studies have demonstrated that filaggrin (FLG) gene polymorphism can directly reduce the structural stability of the stratum corneum, diminish the skin’s natural moisturizing capacity, and render the skin more susceptible to external stimuli (29, 30). Abnormalities in lipid metabolism-related genes, such as CERS3 and UGCG, directly result in insufficient synthesis of core lipids (e.g., ceramides), thereby further weakening the lipid barrier of the stratum corneum (31). Additionally, susceptibility genes including HLA-Cw6 not only participate in the regulation of immune responses but also indirectly interfere with lipid metabolism and epidermal differentiation, thereby further compromising the innate defense capacity of the skin (32–34).

Aberrant expression of epidermal structural proteins is a core feature of barrier defects and exhibits a clear bidirectional regulatory relationship with inflammatory responses (35, 36). Abnormal expression or deficiency of these proteins can directly induce characteristic psoriatic lesions. Downregulated expression of loricrin (LOR) and involucrin (INV) impairs the integrity of the cornified envelope and compromises physical barrier function (37–39). Concurrently, abnormal structural proteins activate keratinocytes, prompting them to secrete pro-inflammatory mediators (e.g., IL-6 and CXCL8), recruit immune cells such as neutrophils and T cells for infiltration, and initiate local inflammatory responses (40–42). In turn, core inflammatory factors, including IL-17 and TNF-α, further inhibit the expression of LOR and INV, forming a vicious cycle (43). This bidirectional regulation ultimately leads to a marked reduction in the skin’s tolerance to external stimuli, triggering the characteristic erythematous and scaly lesions of psoriasis: deficiency of structural proteins causes disorganized arrangement and reduced adhesion of stratum corneum cells, which easily shed to form silvery white scales, while sustained stimulation by inflammatory factors exacerbates scale accumulation and erythematous reactions.

Imbalanced keratin expression also contributes to the pathological process. As markers of normal epidermal differentiation, decreased expression of KRT1/10 exacerbates abnormal keratinocyte differentiation (44–47). In contrast, increased expression of stress keratins (e.g., KRT6/16/17) not only further disrupts epidermal differentiation but also interferes with the expression of lipid-binding proteins, disrupts lipid homeostasis, impairs barrier function, and amplifies inflammatory responses and lesion manifestations.

Lipid metabolism disorder is a core characteristic of skin barrier defects in psoriasis, persisting throughout the entire disease course. It is not merely insufficient lipid synthesis but a comprehensive abnormality involving synthesis, transport, distribution, and homeostatic regulation (48). Ceramides, cholesterol, and fatty acids-core components of the “mortar” in the “brick-and-mortar structure” of the stratum corneum-exhibit metabolic abnormalities that directly affect barrier integrity. Among these, abnormal ceramide synthesis is the most critical, primarily relying on the *de novo* synthesis pathway. Serine palmitoyltransferase (SPT) and ceramide synthases (CERS1-6) are core enzymes in this pathway, with CERS3 and CERS4 being the most significantly inhibited by inflammatory factors (31, 48). Abnormalities in genes such as UGCG and SMPD1 further hinder lipid synthesis and disrupt metabolic homeostasis (31). Epidermal structural proteins and lipid metabolism exert a synergistic effect: aberrant expression of structural proteins (e.g., LOR and INV) impairs the uniform distribution of lipids in the stratum corneum, preventing lipids from properly filling the intercellular spaces. Conversely, insufficient lipid synthesis compromises the stability of structural proteins, further damaging the cornified envelope and forming a synergistic damage cascade: “abnormal structural proteins-disordered lipid distribution-further barrier impairment”.

Tight junctions, a key component of the epidermal barrier, are responsible for maintaining epidermal permeability, and their functional abnormalities are closely linked to lipid metabolism and inflammatory responses (49). Composed of proteins including claudin family members, occludin, and ZO-1 (zonula occludens-1), tight junctions form a dense complex that prevents the invasion of external stimuli, pathogens, and inflammatory mediators while providing a stable internal environment for lipid synthesis and distribution (50–52). In patients with psoriasis, tight junction-related proteins not only show downregulated expression but also ectopic distribution: claudin family proteins and occludin, originally concentrated in the granular layer, are partially shifted to the stratum corneum or basal layer, resulting in a loose complex structure and impaired integrity (49, 51, 53). This abnormality not only provides a pathway for the invasion of pathogens such as *Staphylococcus aureus* and Group A Streptococcus (54), but also hinders intercellular lipid transport, preventing lipids from filling stratum corneum interstices and further weakening the lipid barrier. Simultaneously, inflammatory factors further inhibit the expression of tight junction proteins, exacerbating barrier damage and lipid metabolism disorders, and forming a vicious cycle.

Impaired aquaporin 3 (AQP3) function further exacerbates barrier fragility and is closely associated with lipid metabolism and inflammatory responses (55). AQP3 is mainly expressed in the basolateral membrane of keratinocytes, with its core function being the transmembrane transport of glycerol, water, and small molecules-glycerol is an important raw material for lipid synthesis (56–58). In patients with psoriasis, AQP3 expression is significantly reduced: on the one hand, this leads to insufficient glycerol transport, which not only causes skin dryness and increased transepidermal water loss but also reduces the supply of raw materials for lipid synthesis, directly inhibiting the synthesis of core lipids such as ceramides. On the other hand, the dry microenvironment activates keratinocytes, prompting them to release pro-inflammatory mediators and amplify inflammatory responses. In turn, inflammatory factors further inhibit AQP3 expression and the activity of key lipid synthesis genes, exacerbating lipid disorders and barrier damage (5, 59). Impaired AQP3 function also affects keratinocyte metabolism and differentiation, further interfering with structural protein expression and lipid synthesis, and together with abnormal structural proteins, tight junction damage, and lipid disorders, constitutes the core of barrier defects (60).

Most existing studies focus on abnormalities of a single barrier-related gene or protein; however, psoriasis-related barrier defects exhibit a hierarchical progressive relationship: “genetic susceptibility-structural abnormalities-functional impairment”, with bidirectional feedback between each level. For instance, FLG gene polymorphism not only directly affects stratum corneum moisturization but also indirectly inhibits AQP3 expression by downregulating its transcription factors, forming a gradient damage chain: “gene abnormality-water transport disorder-insufficient lipid synthesis-decreased structural protein stability”. Furthermore, ectopic distribution of tight junctions is not only a manifestation of barrier dysfunction but also reversely regulates keratinocyte differentiation and inflammatory factor secretion by altering the osmotic pressure of the epidermal microenvironment, extending barrier defects from the “structural level” to the “functional level”. This gradient damage mechanism explains why barrier repair in patients with psoriasis requires the simultaneous consideration of gene regulation, structural repair, and functional recovery-simple lipid supplementation or moisturization alone is insufficient to achieve long-term improvement. **Table 1** summarizes the key factors related to skin barrier defects in psoriasis.

**Table 1:** Skin barrier-related indicators in psoriasis.

Constituents	Expression	Function	Roles in psoriasis
KCs	KRT1/10 Downregulation, KRT6/16/17 upregulation	Main cells of stratum corneum, mediate epidermal renewal through keratinization	Excessive proliferation & abnormal differentiation of KCs; hyperkeratosis and parakeratosis
Fliaggrin、Loricrin、Involucrin	Filaggrin/Loricrin downregulation, Involucrin upregulation	Strengthen CE structure, enhance epidermal barrier and maintain skin hydration	Abnormal CE formation, impaired skin barrier, increased antigen permeability
Clauduin1/4/7、ZO-1	Claudin1/7 downregulation, Claudin4/ZO-1 upregulation	Mechanical junction, Permeability barrier, KCs differentiation & proliferation, Signal transduction involvement	IL-1β/TNF-α interaction-induced KCs proliferation; Lymphocyte-activating cytokines
AQP3	AQP3 Downregulation	Transport of water/glycerol/triglyceride, skin barrier maintenance, inflammatory signal transduction involvement	Impaired permeability barrier, inflammatory signaling pathway involvement, amplified inflammation
Cer、 FFA/CHOL	Decreased content or imbalanced proportion of Cer	Enhance skin barrier and maintain hydration status	Increased TEWL, decreased SCH, impaired barrier integrity, activated epidermal innate immunity

### 4.2 Multi-factor driven barrier damage, initiating the pathological process

Barrier defects do not exist in isolation but interact with external stimuli, immune activation, and lipid metabolism abnormalities to initiate the pathological process of psoriasis. External stimuli (e.g., ultraviolet radiation, dryness, chemical irritants) can directly interfere with lipid synthesis, inhibit the expression of lipid-related genes such as CERS3 and CERS4, and reduce ceramide production (31, 61). Concurrently, they exacerbate tight junction damage and structural protein abnormalities, creating conditions for pathogen invasion.

After pathogens such as *Staphylococcus aureus* and Group A Streptococcus invade through the damaged barrier, the toxins and proteases they secrete not only destroy the stratum corneum structure and inhibit structural protein expression but also directly inhibit the activity of key lipid synthesis enzymes (e.g., serine palmitoyltransferase, SPT), damage the lipid matrix structure, and further exacerbate lipid metabolism disorders (54).

Abnormal keratinocyte activation is a key hub in the pathological process. Upon stimulation by barrier defects, lipid abnormalities, and inflammation, keratinocytes undergo sustained abnormal proliferation (44–47). On the one hand, they secrete proteases that further damage structural proteins, tight junctions, and lipid-binding proteins, disrupting lipid homeostasis. On the other hand, they release pro-inflammatory factors that recruit immune cell infiltration, activate systemic immune responses, and induce the secretion of core inflammatory factors such as IL-17, TNF-α, and IFN-γ. These inflammatory factors exacerbate barrier damage and lipid disorders through two pathways: first, they directly inhibit the expression of lipid synthesis-related genes such as CERS3 and CERS4, reduce lipid synthase activity, and decrease the production of core lipids such as ceramides and cholesterol (31, 61). Second, they inhibit the expression of structural proteins (e.g., FLG, LOR), tight junction proteins, and AQP3, further impairing barrier function, thus forming the initial cycle: “barrier damage-keratinocyte activation-inflammation activation-further barrier and lipid damage”.

External stimuli in psoriasis act via a “threshold down-regulation effect” rather than simple induction. Traditionally viewed as inducers, they actually lower the disease onset threshold. Patients with congenital skin barrier defects have reduced stimulus tolerance. Stimuli (UV, dryness, etc.) do not directly trigger psoriasis but inhibit lipid synthases (e.g., SPT) and degrade tight junctions, worsening barrier dysfunction. Once damage exceeds the pathological threshold, keratinocyte activation and inflammation ensue. Notably, distinct stimuli exert synergistic damaging effects (e.g., dryness amplifies UV injury; irritants promote pathogen invasion), accelerating the pathogenic cycle. Clinically, prevention should rely on personalized, multi-dimensional stimulus avoidance tailored to individual barrier weaknesses, not just single-stimulus restriction.

### 4.3 Inflammation reversely damages the barrier and strengthens the pathological cycle

Once initiated, the inflammatory response reversely damages the skin barrier and lipid homeostasis through multiple pathways, strengthens the pathological cycle, and promotes the chronicity of psoriasis. Core inflammatory factors (IL-17, TNF-α, IFN-γ) continuously inhibit structural protein expression, further impairing the integrity of the cornified envelope. Simultaneously, they persistently suppress the activity of key lipid synthesis genes, reduce ceramide synthesis, disrupt lipid distribution and transport, and weaken the moisturizing and defensive functions of the lipid barrier (31, 61).

Inflammation further exacerbates tight junction damage, leading to sustained increases in epidermal permeability and continuous invasion of pathogens and inflammatory factors, forming a secondary cycle: “inflammation-tight junction damage-pathogen invasion-lipid damage-inflammation exacerbation” (54). Additionally, inflammation inhibits AQP3-mediated glycerol transport, further reducing the supply of raw materials for lipid synthesis and exacerbating skin dryness and lipid disorders (56–58). It also interferes with normal keratinocyte differentiation, preventing them from effectively participating in lipid synthesis and secretion, and further compromising lipid barrier function.

It is important to clarify that lipid metabolism disorder is an important intermediate link between inflammation and barrier defects, rather than the core driving factor of the disease. It is intertwined with abnormal epidermal structural proteins, tight junction damage, impaired AQP3 function, and keratinocyte activation to maintain the pathological state. Insufficient ceramide synthesis not only weakens the lipid barrier but also amplifies IL-17-related inflammation by activating CD1a-mediated immune responses (31), further promoting the pathological process.

**Figure 2:**
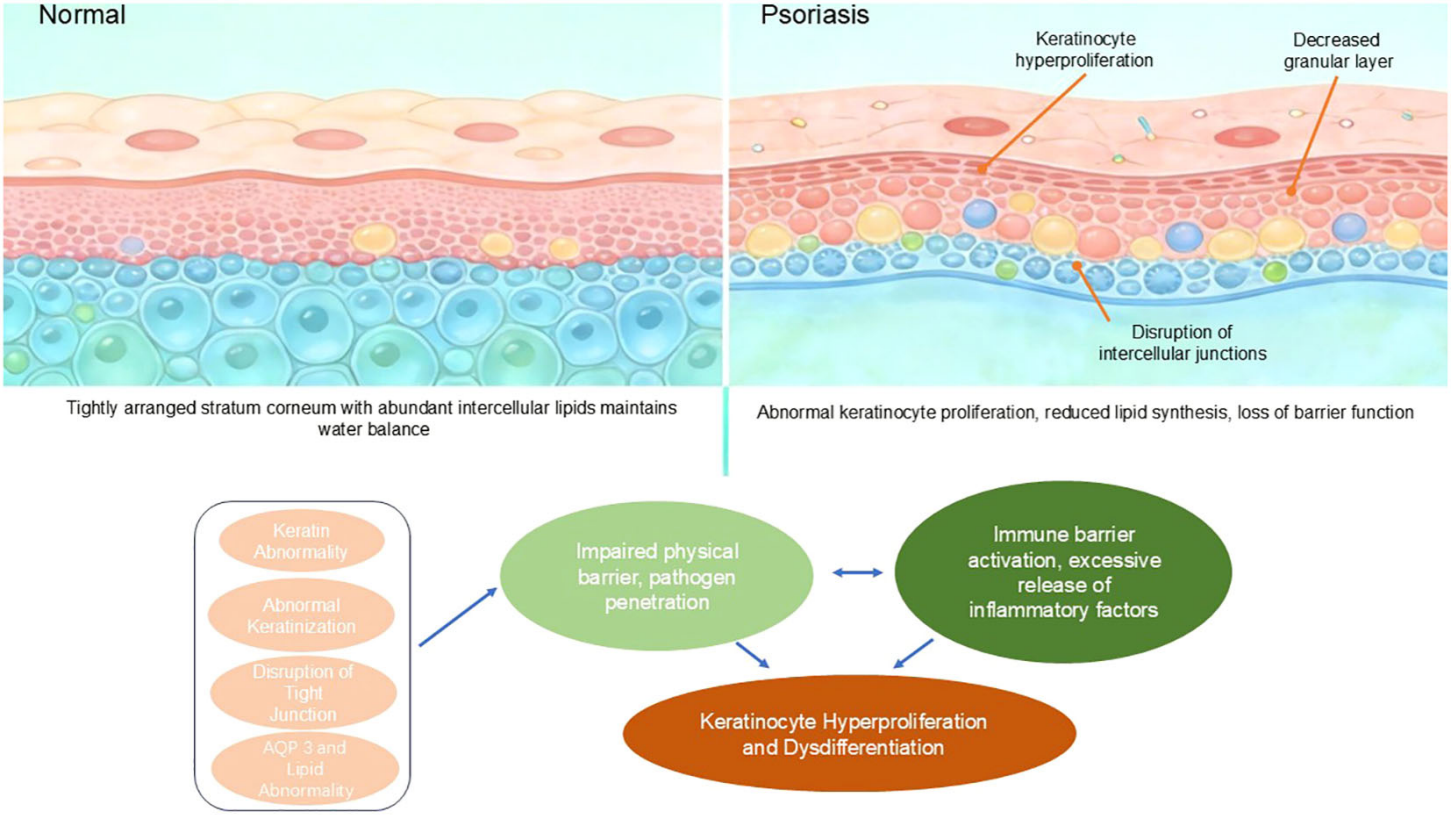
Multifactorial mediation of skin barrier impairment in psoriasis. Under physiological conditions, the skin barrier functions intactly; its impairment in psoriasis is mediated by the synergistic effects of multiple factors. Abnormal KCs activity, dysregulated skin structural and lipid metabolism, disrupted epidermal intercellular junctions, hyperactivation of proinflammatory cytokines, and genetic/environmental factors collectively damage the skin barrier and trigger a pathological vicious cycle.

The aforementioned factors interact to form a complete pathological loop. Abnormal keratinocyte activation, as the core hub of this loop, is not only affected by various factors but also further damages the barrier, lipids, and AQP3 function by releasing pro-inflammatory factors and proteases, strengthening the pathological cycle. This highlights the important value of skin moisturization and barrier repair in the adjuvant treatment of psoriasis. Clinical interventions can repair structural proteins, tight junctions, and AQP3 function, supplement core lipids such as ceramides, restore lipid homeostasis, and break the pathological cycle, thereby improving lesion symptoms and reducing disease recurrence. **Figure 2**, **3** illustrate the molecular mechanistic models underlying skin barrier impairment in psoriasis.

**Figure 3:**
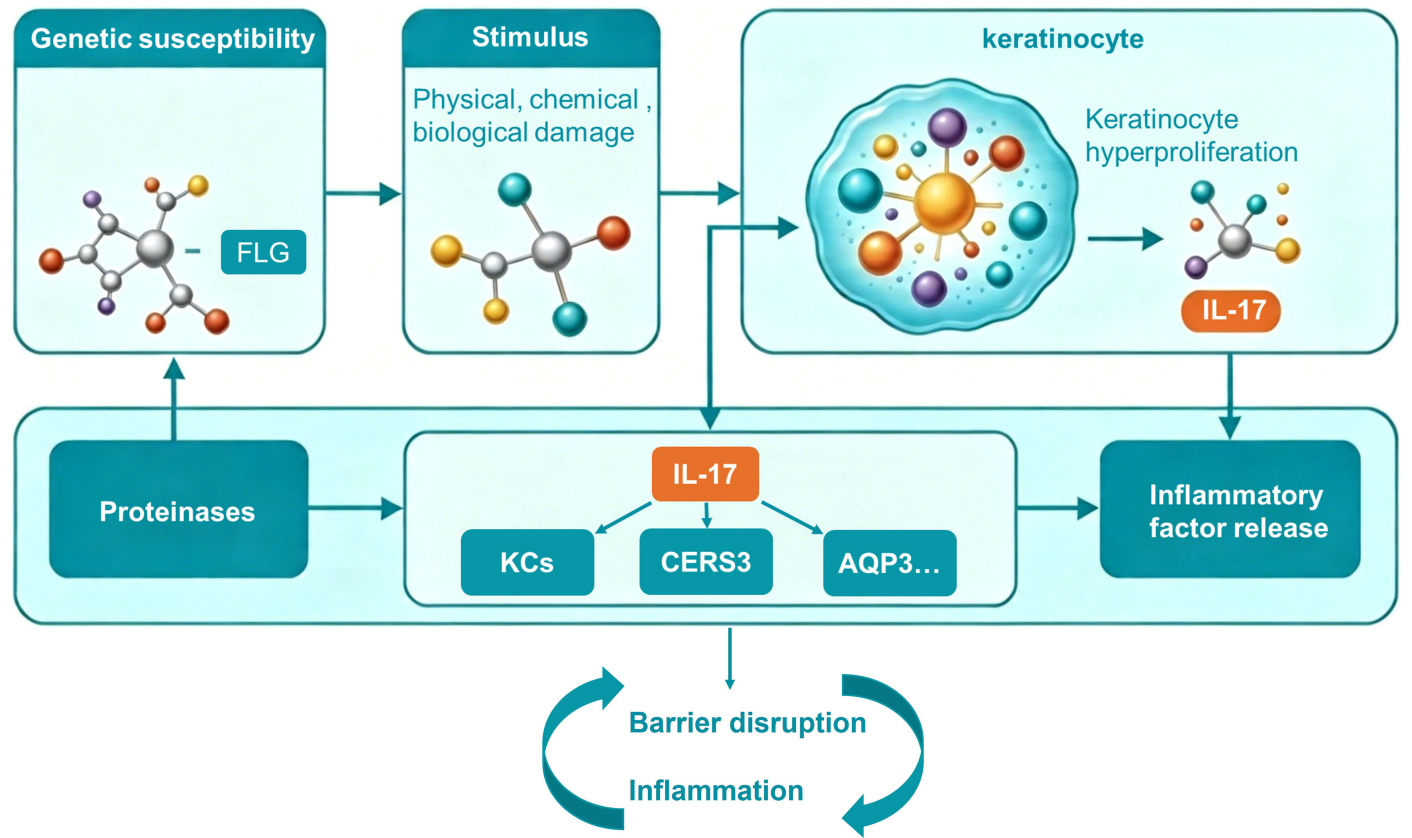
Schematic diagram showing the vicious cycle of skin barrier impairment and inflammation in psoriasis. Genetic susceptibility (e.g., FLG deficiency) and external stimuli induce barrier damage, which activates keratinocytes to secrete pro-inflammatory factors (e.g., IL−17). These factors further suppress barrier-associated proteins (e.g., Claudin, Occludin), exacerbating barrier dysfunction and sustaining inflammation, ultimately leading to psoriatic lesions.

## 5 The significance of skin barrier repair in clinical treatment of psoriasis

Impaired skin barrier function is closely correlated with the clinical manifestations and prognosis of psoriasis, including disease severity, seasonal exacerbation, and treatment resistance-factors that directly impact patients’ quality of life and treatment outcomes. Psoriatic lesions are typically characterized by reduced epidermal water content, depleted natural moisturizing factors, and decreased free fatty acids; notably, regions with inherently compromised barrier function (e.g., the anterior tibia) often develop refractory lesions that are unresponsive to conventional therapeutic regimens. Seasonal fluctuations in disease activity, with exacerbation in cold winter and spring and improvement in warm summer, are primarily associated with reduced ceramide levels in the stratum corneum under cold conditions, which directly disrupts barrier integrity and triggers disease flare-ups-a finding consistent with the mechanism elaborated in Section 4, wherein abnormal ceramide synthesis is identified as a core feature of skin barrier impairment (5, 62).

Clinical practice and evidence-based research have confirmed that lipid-based topical interventions serve as the cornerstone of skin barrier repair, with their value extending beyond symptom alleviation to include optimized therapeutic efficacy and reduced disease recurrence-advantages that complement the mechanistic insights presented in Section 4. Specifically, ceramide-based interventions, categorized into monotherapy and combination regimens, have been validated in multiple clinical trials. In a single-intervention study, two cohorts of 30 and 60 patients received unilateral application of a ceramide-containing moisturizer; the proportions of patients with delayed disease recurrence in the 20-day and 30-day treatment groups were 54.5% and 71%, respectively, confirming its efficacy in preventing acute disease episodes (60). In a randomized controlled trial (RCT) involving 106 patients with psoriasis, the group treated with a linoleic acid-ceramide moisturizer combined with 0.1% mometasone furoate cream (Group T1) demonstrated superior improvement in PASI-50 at week 8, earlier reduction in transepidermal water loss (TEWL) of lesions, and higher skin hydration levels compared to the group treated with 0.1% mometasone furoate cream alone (Group C1). Furthermore, during 1 year of continuous treatment, Group T2 (maintaining the combination regimen) exhibited a lower recurrence rate and more stable lesion-related indices than Group C2 (discontinuing the moisturizer) (8). Another multicenter RCT involving 178 patients further confirmed that 4-week treatment with mometasone furoate combined with a linoleic acid-ceramide-containing moisturizer effectively reduced the recurrence rate of psoriasis, consistent with the aforementioned findings (63). Additionally, ceramide-phospholipid composite liposomes have emerged as a novel therapeutic approach, capable of enhancing the lipid bilayer, promoting drug penetration, and downregulating pro-inflammatory cytokines, thereby achieving a synergistic effect of barrier repair and anti-inflammatory activity (64).

Consistent with the Chinese Guidelines for Psoriasis, emollient-based barrier repair has become an indispensable adjuvant therapy for psoriasis, playing a pivotal role in reducing the frequency and severity of disease recurrence (65). Building on the pathological mechanisms elaborated in Section 4, targeted barrier repair strategies have been developed beyond mere moisturization, including the regulation of structural protein expression (filaggrin, loricrin), stabilization of tight junctions (Claudin-1/7), modulation of AQP3 function, and supplementation of ceramides, cholesterol, and free fatty acids at optimal ratios. Importantly, non-invasive indices such as TEWL and skin hydration can serve as objective biomarkers for disease assessment, therapeutic monitoring, and prognosis prediction, providing a scientific basis for personalized barrier repair. Collectively, these findings underscore the crucial clinical value of skin barrier repair in the comprehensive management of psoriasis, bridging the gap between basic mechanistic research and clinical practice.

## 6 Conclusion

In summary, this review highlights that skin barrier dysfunction serves as a pivotal contributor to the pathogenesis of psoriasis, with a robust bidirectional interplay between epidermal barrier impairment and psoriatic inflammation forming a self-reinforcing vicious cycle that drives disease initiation, progression, and chronic recurrence. Notably, disrupted skin barrier function represents a shared pathological feature across psoriasis, atopic dermatitis (AD), and other barrier-related skin disorders. However, these conditions differ substantially in their etiological origins, molecular mechanisms, and clinical phenotypes, while also sharing an overlapping barrier–inflammation regulatory axis, providing important implications for both mechanistic exploration and clinical intervention.

The key distinctions between psoriasis and other barrier-associated diseases, particularly AD, can be summarized as follows. First, with regard to the origin of barrier damage, AD is primarily driven by congenital structural abnormalities, most notably pathogenic mutations in filaggrin (FLG), where barrier defects typically precede inflammatory activation. In contrast, barrier dysfunction in psoriasis is predominantly a secondary event driven by chronic inflammation, with inflammatory signals such as IL-17 and IL-22 dominatingly suppressing keratinocyte differentiation, in the absence of typical FLG loss-of-function mutations. Second, at the structural level, AD is characterized by marked downregulation of FLG, loricrin (LOR), and natural moisturizing factors (NMF), accompanied by severely elevated transepidermal water loss (TEWL). Psoriasis, by contrast, exhibits prominent keratinocyte hyperproliferation and parakeratosis, along with disordered structural protein expression and aberrant upregulation of stress-induced keratins including KRT6/16/17. Third, regarding lipid barrier abnormalities, AD mainly presents reduced total ceramide content and imbalanced lipid ratios, whereas psoriasis is distinguished by disturbed ceramide subclass composition rather than simply global ceramide depletion. Finally, in terms of immune polarization, AD is primarily driven by a Th2/IL−4/IL−13 axis, whereas psoriasis is defined by a dominant Th17/IL−17/IL−22 signature (66, 67). Of note, both psoriasis and acne share Th17−mediated inflammatory profiles. Other conditions such as ichthyosis vulgaris and allergic contact dermatitis display distinct barrier disruption mechanisms and lack the Th17−skewed immunity and keratinocyte hyperproliferation characteristic of psoriasis (68).

Clinically, these mechanistic differences carry important therapeutic implications: early and intensive barrier repair represents a cornerstone in AD management, whereas controlling core inflammatory pathways is central to psoriasis treatment. Nonetheless, the precise regulatory networks governing the crosstalk between the skin barrier and psoriasis inflammation, as well as standardized clinical protocols for barrier-targeted adjuvant therapy, remain to be fully elucidated. Future research should prioritize the identification of barrier-related novel molecular targets, the development of sensitive and noninvasive detection methods, the establishment of unified clinical guidelines, and the design of innovative barrier-directed therapeutic strategies. These efforts will help establish a solid foundation for the precise diagnosis, risk stratification, and personalized management of psoriasis and other immune-mediated barrier-related skin diseases.
